# Supplementary figures and images for: Maternal supplementation with Bifidobacterium breve M-16V prevents their offspring from allergic airway inflammation accelerated by the prenatal exposure to an air pollutant aerosol
Source: PLoS One. 2020 Sep 11;15(9):e0238923. doi: 10.1371/journal.pone.0238923 (PMC7485856; doi:10.1371/journal.pone.0238923)

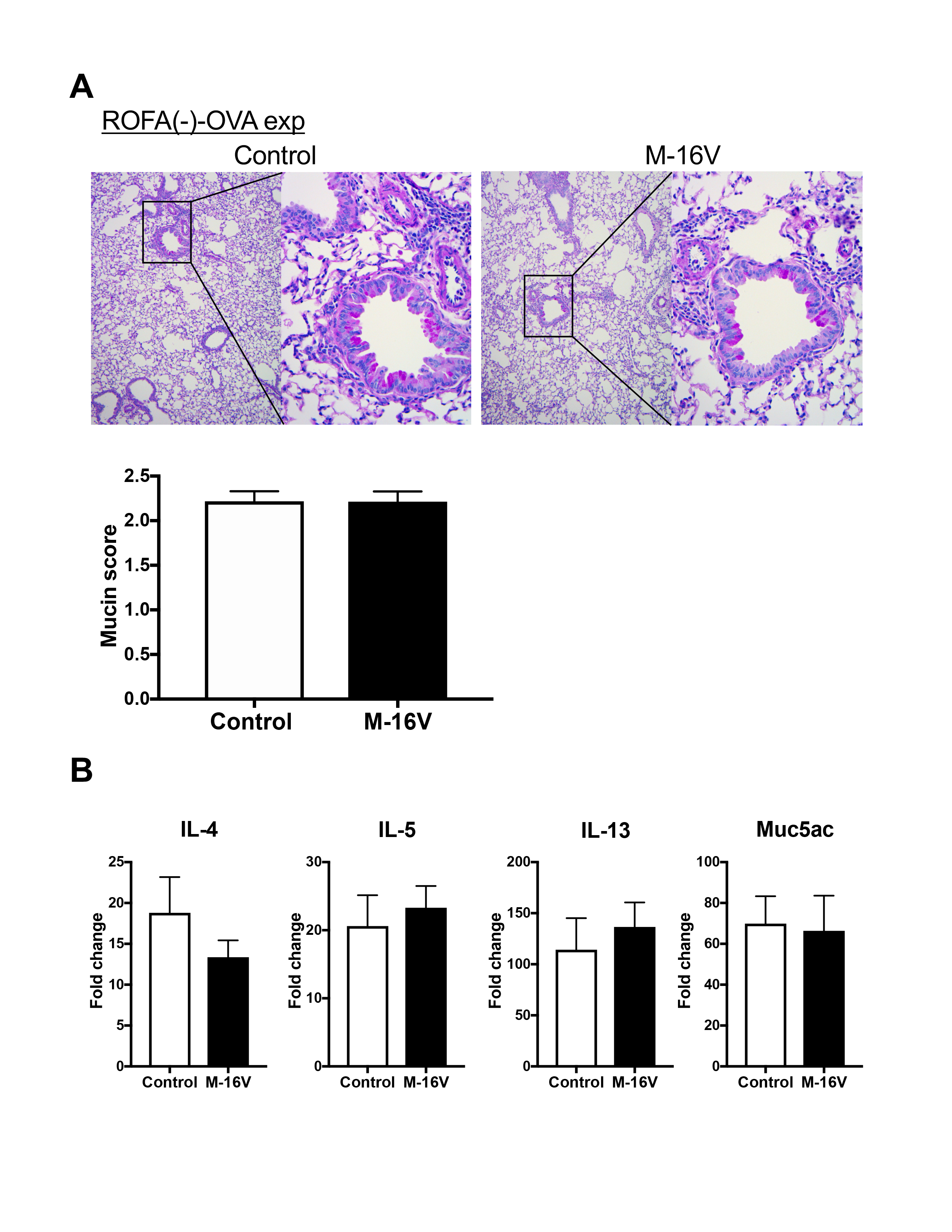

Supplement: S1 Fig — (A) Lung sections were stained with PAS to visualize mucus production at day 30 of life. (B) Quantitative analysis of mucus production using a scoring system of 1–4 detailed in the Materials and methods. The values are presented as the means ± SEM (n = 6–8). (TIF) [file pone.0238923.s001.tif]

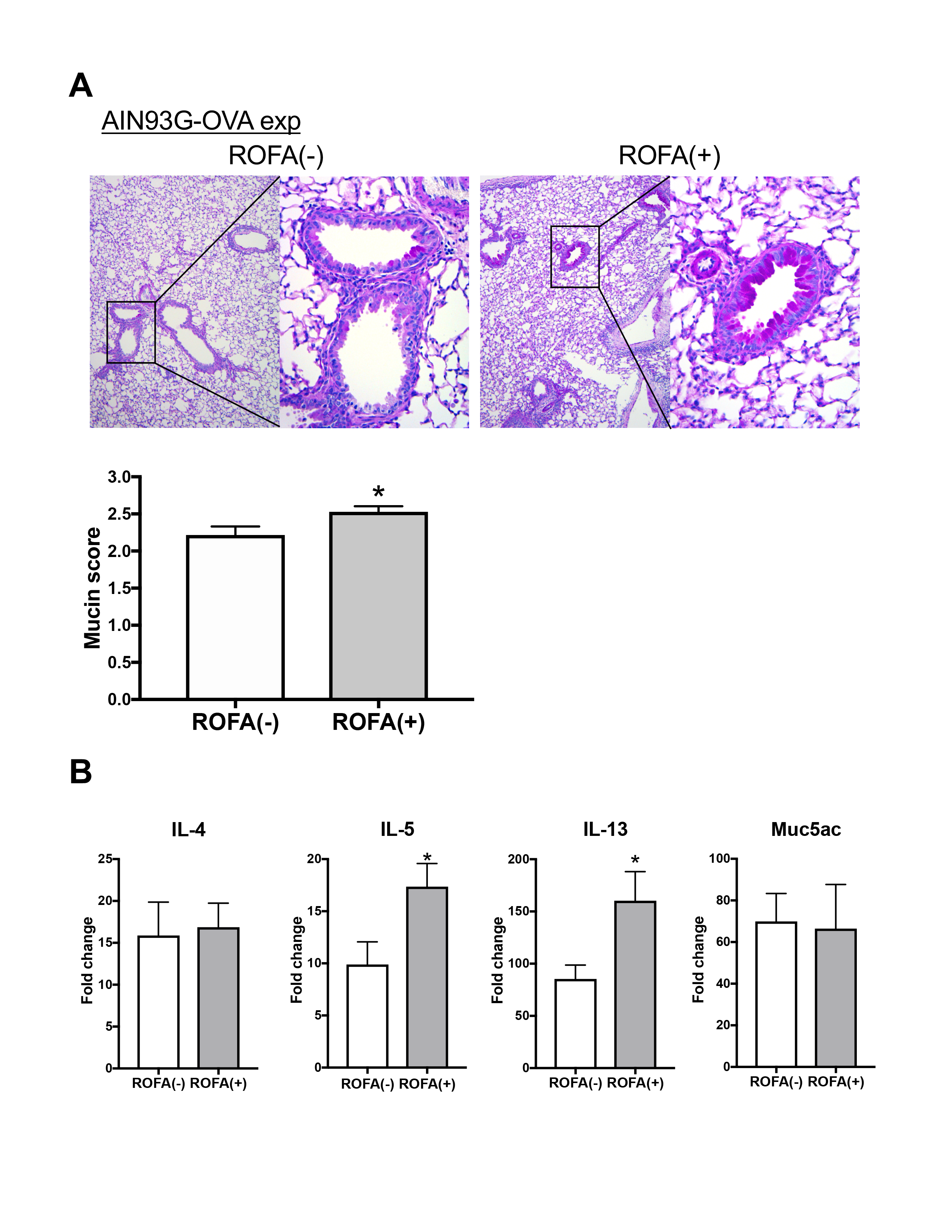

Supplement: S2 Fig — (A) Lung sections were stained with PAS to visualize mucus production at day 30 of life. (B) Quantitative analysis of mucus production using a scoring system of 1–4 detailed in the Materials and methods. The values are presented as the means ± SEM (n = 6–7). *P < 0.05 compared with OVA-sensitized and -exposed neonates from mothers without ROFA exposure. (TIF) [file pone.0238923.s002.tif]

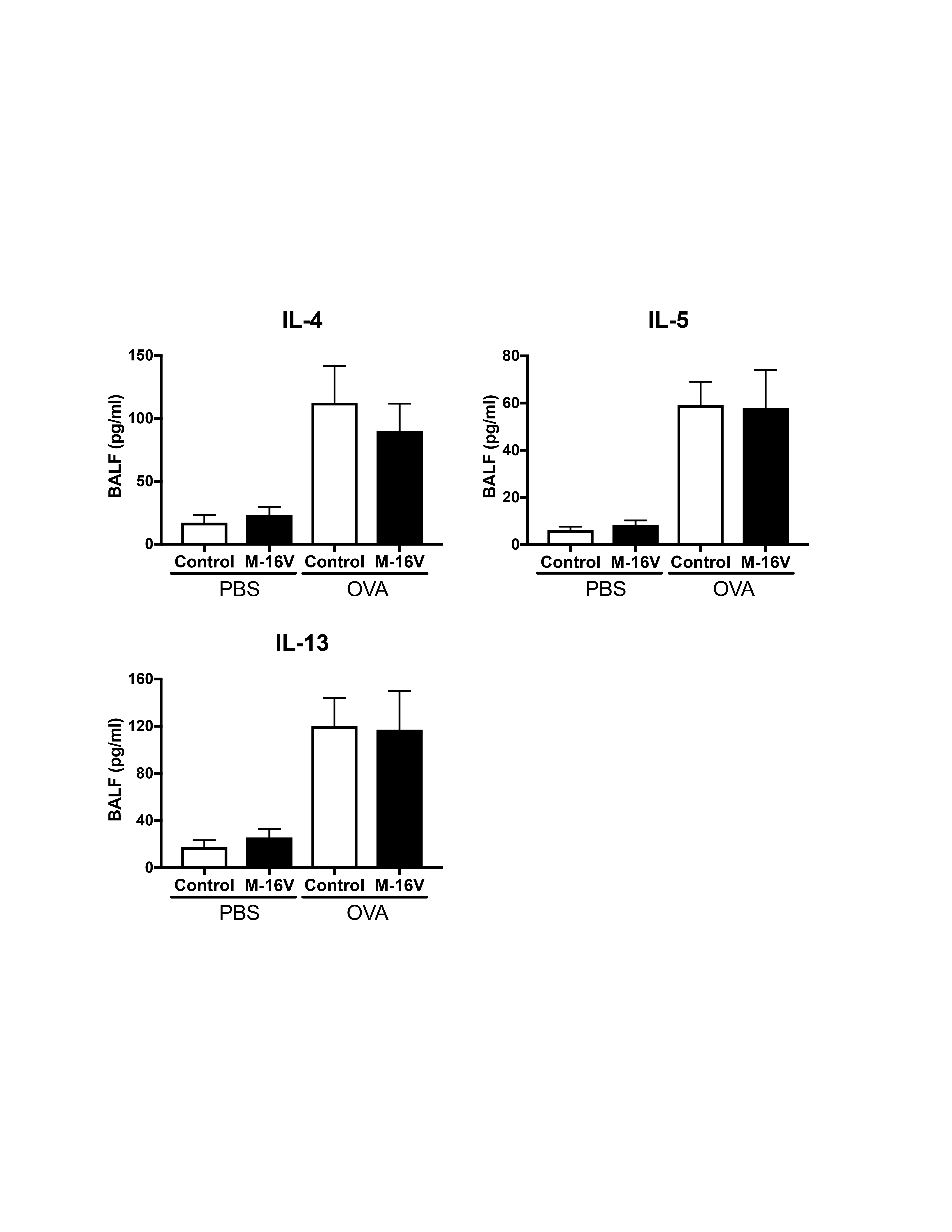

Supplement: S3 Fig — BALF was collected 48 h after the final aerosol challenge, and BALF supernatant was collected after the centrifugation of samples at 800 ×g for 5 min. Cytokine productions of IL-4, IL-5, and IL-13 from BALF supernatants were measured by ELISA. The values are presented as the mean ± SEM (n = 6–21). (TIF) [file pone.0238923.s003.tif]
