# Supplementary material for: Maternal supplementation with Bifidobacterium breve M-16V prevents their offspring from allergic airway inflammation accelerated by the prenatal exposure to an air pollutant aerosol
Source: PLoS One. 2020 Sep 11;15(9):e0238923. doi: 10.1371/journal.pone.0238923 (PMC7485856; doi:10.1371/journal.pone.0238923)
Supplement: Attachment — Submitted filename: Response to reviewer-2.docx [file pone.0238923.s005.docx]

**Ms # PONE-D-20-05759R1**

**Response to Reviewer comment**

**Reviewer #1:**

**Some final comments:**

We again appreciate the comments and suggestions by Reviewer #1, and we have addressed each with additional comments.

**1. Lines 337-339: The sentence sounds incomplete. Please check.**

Thank you for the suggestion. The sentence was incomplete. We changed the sentence as follows; “It is reported that *Streptococcus* and *Blautia* produce substances such as bacteriocins, enzymes, lactic acid, and fatty acids.”

**2. Line 347: The wording "improved allergic responses" can be misleading. Better use "reduced allergic responses".**

We now use "reduced allergic responses" as suggested.

**Reviewer #2:**

**Most of my comments were addressed. However, I feel it reads a bit complicated now, so I have the following suggestions:**

We again appreciate the comments and suggestions by Reviewer #2, and we have addressed each with additional comments.

**1. Change the title to a more simplistic one - it is hard to get the message**

Thank you for the suggestion. We changed the title as follows to send the message for readers; “Maternal supplementation with *Bifidobacterium breve* M-16V prevents their offspring from allergic airway inflammation accelerated by the prenatal exposure to an air pollutant aerosol”.

**2. The additional first paragraphs in the results section confuse the reader on what was done in the following experiments. State clearly which mice where used for experiments and what exposure was applied. Say that only ROFA exposed mice were used later and that this was the focus.**

We modified Figure 1 to state clearly which mice where used for experiments and what exposure was applied. Also, we now say that only ROFA exposed mice were used later and that this was the focus in this study (L221).

**3. Accordingly, Figure 1 is misleading as it suggests all groups have been investigated throughout the experiment - please communicate the experimental setup clearly to the reader.**

We modified Figure 1 together with comment #2 to communicate the experimental setup clearly to the reader.
